# Changes in physical activity levels and mental health during COVID-19: Prospective findings among adult twin pairs

**DOI:** 10.1371/journal.pone.0260218

**Published:** 2021-11-22

**Authors:** Glen E. Duncan, Ally R. Avery, Siny Tsang, Bethany D. Williams, Edmund Seto

**Affiliations:** 1 Department of Nutrition and Exercise Physiology, Washington State University Health Sciences Spokane, Spokane, Washington, United States of America; 2 Department of Environmental and Occupational Health Sciences, University of Washington, Seattle, Washington, United States of America; University of the West Indies at Saint Augustine, TRINIDAD AND TOBAGO

## Abstract

**Background:**

Physical distancing and other COVID-19 pandemic mitigation strategies have negatively impacted physical activity (PA) levels and mental health in cross-sectional studies. The purpose of this study was to investigate associations between changes in PA and mental health outcomes during the COVID-19 pandemic, following implementation of mitigation strategies, in a sample of adult twins.

**Methods:**

This was a prospective study of 3,057 adult twins from the Washington State Twin Registry. Study participants completed online surveys in 2020, at baseline (March 26 –April 5), and three follow-up waves (W1: April 20 –May 3; W2: Jul 16 –Aug 2; W3: Sept 16 –Oct 1). Physical activity was operationalized as self-reported moderate-to-vigorous PA (MVPA) and neighborhood walking (minutes/week), and mental health outcomes, operationalized as self-reported anxiety and perceived stress were assessed in the three waves of follow-up. Latent growth curve models (LGCMs) were used to assess changes in PA and mental health outcomes over time. Parallel LGCMs were used to estimate the cross-sectional, parallel, and prospective associations between PA and mental health over time. All models took into within-pair correlations and adjusted for age, sex, and race.

**Results:**

Individuals’ amount of MVPA and walking decreased over time, whereas levels of anxiety remained stable, and stress increased slightly. Cross-sectional associations observed between both PA predictors and mental health outcomes were weak. After taking into account cross-sectional associations between PA and mental health outcomes, changes in PA over time were not associated with changes in mental health outcomes over time.

**Conclusions:**

Over a time period aligned with COVID-19 mitigation strategies and social restrictions, changes in physical activity was not associated with changes in anxiety or stress levels in the current sample. Nonetheless, the average decline in PA over time is worrisome. Public health resources should continue to promote PA as a means to improve physical health during the pandemic.

## Introduction

The novel coronavirus (severe acute respiratory syndrome coronavirus 2 (SARS-CoV-2) virus and associated disease COVID-19, abbreviated as COVID-19 throughout) continues to be a major global health concern [1]. Since the first documented appearance of the virus in Wuhan, China in December 2019, there have been over 106 million documented cases worldwide, including over 26 million cases and 464,412 attributable deaths in the United States (U.S.) as of February 11, 2021 [2]. In response to the outbreak, mitigation strategies including physical distancing, closing of businesses and activity spaces (e.g., restaurants, schools, gyms, etc.), and recommendations to wear masks have been enforced nationwide to slow rates of infection and prevent mortality [3]. While effective in preventing the spread of COVID-19 [4], there is concern that such strategies and their inherent effects on daily life may negatively impact mental health. Given that social isolation and/or fear of contracting the virus itself may have a negative impact on mental health [5], it is crucial to identify modifiable factors to potentially improve such outcomes.

Adequate levels of moderate-to-vigorous physical activity (MVPA) and leisure-time walking are related to positive mental health outcomes [6, 7] in adults. Specifically, higher physical activity levels are related to lower severity and prevalence of anxiety and stress disorders through hypothesized psychological and neurobiological mechanisms, including increased self-efficacy and central transmission of norepinephrine [6–10]. Regardless of these known benefits, 52% of U.S. adults do not meet recommendations for aerobic activity [11, 12], and on average, achieved fewer than 5,000 steps/day [13], prior to the COVID-19 outbreak. When considering these concerns within the COVID-19 context, restrictions and social distancing may create additional barriers to physical activity participation through closures of important activity-promoting businesses and time limitations due to increased child-care and/or household responsibilities. Decreases in physical activity resulting from COVID-19 restrictions could further exacerbate negative health outcomes, including mental health and overall well-being. While a limited number of studies showed that reduced physical activity is associated with anxiety- and stress-related symptoms since the onset of COVID-19 [14–16], most of these studies are cross-sectional. Longitudinal studies are needed to understand mental health outcomes in relation to COVID-19 over a timeline of fluctuating restrictions to inform appropriate community resources to promote health and well-being during the pandemic.

Studies in multiple countries, including the U.S., report weight gain and decreases in physical activity after community-wide implementation of COVID-19 mitigation strategies [17–19]. For example, among a sample of 909 twin pairs (77% identical) primarily living in the U.S., a cross-sectional study by our group reported that 42% of study participants reported a decrease in physical activity levels in the two-weeks after COVID-19 restrictions were implemented statewide (i.e., March 2020) [20]. Perceived decrease in physical activity was associated with higher stress levels, but the association was confounded by between-family influences shared between twins from the same family. This means that stress levels did not differ between a pair of identical twins with different perceived change in physical activity (i.e., one twin reported no change, whereas their co-twin reported decrease). On the other hand, the association between perceived decrease in physical activity and anxiety was mediated by between-family influences, though the effect was small and confounded by demographic factors. This means that, between a pair of identical twins, anxiety level was higher for the co-twin who reported less physical activity, as compared to their co-twin who reported no change in physical activity. As identical twins raised together share 100% of their genes and have similar familial background, the observed association between perceived decrease in physical activity and anxiety after controlling for between-family confounds can be considered “quasi-causal” [21]. Due to the cross-sectional nature of our previous study, it remains unclear whether the association between physical activity and mental health outcomes persists and/or changes over time as society navigates the evolving COVID-19 outbreak and related mitigating strategies.

Over the course of 2020, spread of COVID-19, and resulting mandates and restrictions, have fluctuated and influenced resident’s daily lives. At the beginning of March 2020, Washington state had the highest number of confirmed cases per capita of any state in the U.S. [2], and the state’s resulting stay-home order was issued March 23rd [2]. Incidence of new cases began to decline overall with some phased re-openings beginning in some counties two to three months later (May/June 2020), then as cases continued to increase into the Fall season more stringent restrictions were re-implemented in November 2020 [2]. Given the cross-sectional nature of our previous work, a follow-up is essential to understand how physical activity levels have been impacted over time as COVID-19 restrictions have evolved, and whether these changes are associated with mental health outcomes over the medium to longer term. The objective of the current study was to investigate the association between changes in physical activity (i.e., MVPA and walking) and changes in mental health outcomes (i.e., stress and anxiety) over three-waves of data collection, representing approximately a six-month span. Twins who completed our prior cross-sectional study were recruited to complete follow-up surveys. While the current study took into account within-pair correlations, the extent to which between-family confounds mediated the association between physical activity and mental health was not addressed due to limited sample size. We hypothesized that decreases in physical activity would be associated with lower levels and/or decreases in mental health outcomes over time.

## Materials and methods

### Participants

Data for the current study was obtained from a sample of twins from the Washington State Twin Registry (WSTR) who participated in a series of online surveys examining the impact of COVID-19 mitigation on a number of health-related behaviors and outcomes over the first seven months of the pandemic. The WSTR is a community-based Registry of twin pairs primarily recruited through Washington State Department of Licensing (DOL) records. Details about the WSTR’s prior recruitment procedures and additional information are reported elsewhere [22–24]. This study was approved by the Washington State University Institutional Review Board. Consent was assumed upon completion of the online REDCap questionnaire [25]. All WSTR members with an email address were invited to participate in the baseline survey; survey participation was voluntary, and remuneration was not provided.

Baseline surveys were completed in March 2020 by 3,791 twins (909 pairs) as reported by us previously [20]. Invitations to participate in the follow-up surveys were only sent to those who completed the baseline survey. To date, three waves of follow-up surveys were administered (W1: April 20 –May 3, *n* = 3,057; W2: Jul 16 –Aug 2, *n* = 2,608; W3: Sept 16 –Oct 1, *n* = 2,491); retention was over 60% across the three follow-up surveys. The baseline survey assessed perceived change in physical activity over the two-weeks following implementation of mitigation strategies (decreased a lot, decreased somewhat, no change, increased somewhat, increased a lot). In contrast, each of the follow-up surveys collected information on self-reported amount of physical activity (described below). The current study focused only on data collected in the three waves of follow-up (W1-W3) for physical activity and mental health outcomes (anxiety and stress).

### Measures

#### Physical activity

Physical activity was operationalized as weekly MVPA and neighborhood walking. Participants reported, in the past two weeks, the number of days per week they engaged in moderate physical activity for at least 30 minutes and vigorous physical activity for at least 20 minutes. The amount of MVPA (minutes per week) was created by summing the moderate and vigorous physical activity days by their respective durations. This measure has been validated with objective physical activity data measured using accelerometers and GPS data loggers within a subsample of the WSTR (*n* = 280, r = 0.48, 95% CI = 0.39–0.57, *p* < 0.001) [26, 27].

For neighborhood walking, participants reported, in the past two weeks, how many days during a typical week they walked in their neighborhood, with a follow-up question about the average number of minutes spent in each walking bout [28, 29]. Responses of less than 15 minutes were coded as 10 minutes, whereas responses of 90 or more minutes were top coded as 90 minutes. Items on frequency (number of days) and duration were multiplied to obtain the total amount of walking (minutes per week). Neighborhood walking has been validated against objective data (*r* = 0.47, 95% CI = 0.38–0.56, *p* < 0.001) and correspond to health recommendations [28, 29].

#### Mental health

Anxiety was assessed using the six-item anxiety subscale in the Brief Symptom Inventory [BSI; 30]. Participants were asked to indicate how much discomfort each of six potential “problems” has caused them during the past two weeks (0 = Not at all; 1 = A little bit; 2 = Moderately; 3 = Quite a bit; 4 = Extremely). Example “problems” included nervousness/shakiness, feeling suddenly scared, and feeling tense or keyed up. The total summed anxiety score ranges from 0 to 24, with higher scores reflecting higher levels of anxiety.

Stress was assessed using the 10-item Perceived Stress Scale [PSS; 31]. Participants were asked about their feelings and thoughts in the last two weeks (0 = Never; 1 = Almost never; 2 = Sometimes; 3 = Fairly often; 4 = Very often). Examples of feelings and thoughts they may have experienced included frequency of being upset, feeling unable to control important things in life, and feeling nervous and “stressed”. The total PSS score ranges from 0 to 40, with higher scores reflecting higher levels of perceived stress.

#### Covariates

Participants’ age, sex, and race were included as covariates in the statistical analyses. Age referred to individuals’ age at which they completed the baseline survey, computed based on their reported date of birth upon enrollment with the WSTR. Sex was self-reported as male or female. Race was assessed by participants’ response to whether they considered themselves to be one or more of the six response categories (White, American Indian or Alaska Native, Black or African-American, Native Hawaiian or Pacific Islander, Asian, and/or Other). In the current sample, due to high prevalence of White respondents (~96%) participants were coded as White or non-White.

### Statistical analysis

We computed Pearson correlations between MVPA/walking and the two mental health indicators (anxiety and stress) separately for W1, W2, and W3. These estimates reflect the cross-sectional association between the two activity predictors and two mental health outcomes at each wave of the follow-up surveys, without considering correlations within twin pairs and/or within-person correlations.

**Fig 1:**
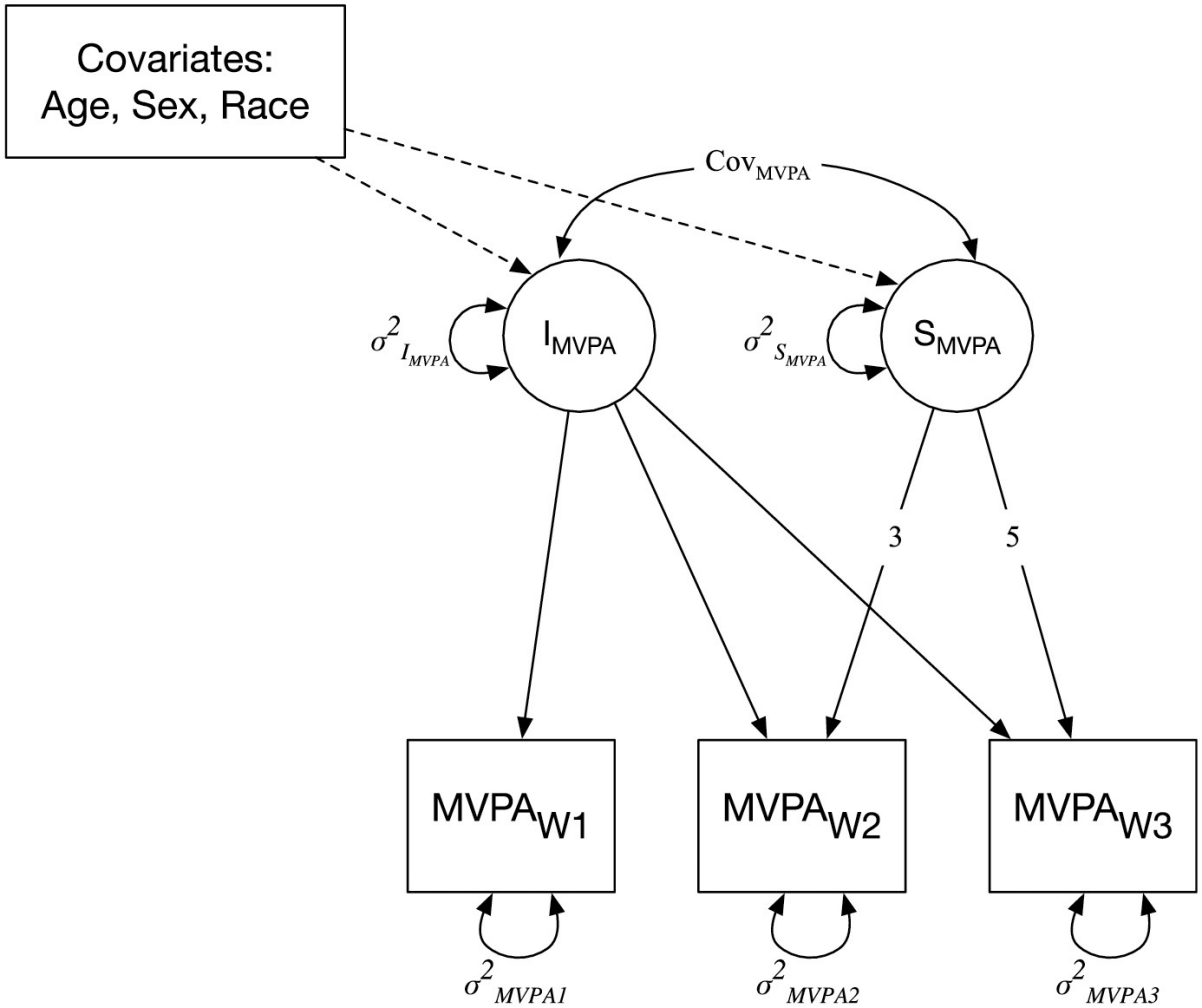
Latent growth curve model estimating the change in moderate-to-vigorous physical activity (MVPA) from follow-up surveys W1 to W3. I = latent intercept, representing the average level of MVPA at W1. S = latent slope, reflecting the average change in MVPA from W1 to W3. Cov_MVPA_ = covariance between the latent intercept and slope. MVPA = moderate-to-vigorous-physical activity. W1-W3 = follow-up survey waves 1 to 3. *Note*. The growth parameters are constrained to 0 (illustrated with the lack of arrow), 3, and 5 to illustrate the number of months between W1, W2, and W3.

Four latent growth curve models (LGCMs) were used to estimate the change in walking, MVPA, anxiety, and perceived stress from W1 to W3 (Fig 1). LGCM is a multivariate statistical method that allows for the estimation of inter-individual heterogeneity in change over time [32]. For each LGCM, the latent intercept (I) represents the average levels of MVPA, walking, anxiety, and stress, respectively at the first follow-up survey (W1), and the latent slope (S) reflects the average change across the three waves of assessments (W1 to W3) for all participants. A positive latent slope reflects an increase in the outcome variable, whereas a negative slope indicates a decrease in the outcome variable from W1 to W3. The covariance between the latent intercept and slope (Cov_(*I*,*S*)_) is also estimated, which reflects the extent to which the initial levels of activity or mental health outcomes are associated with the corresponding change over time.

**Fig 2:**
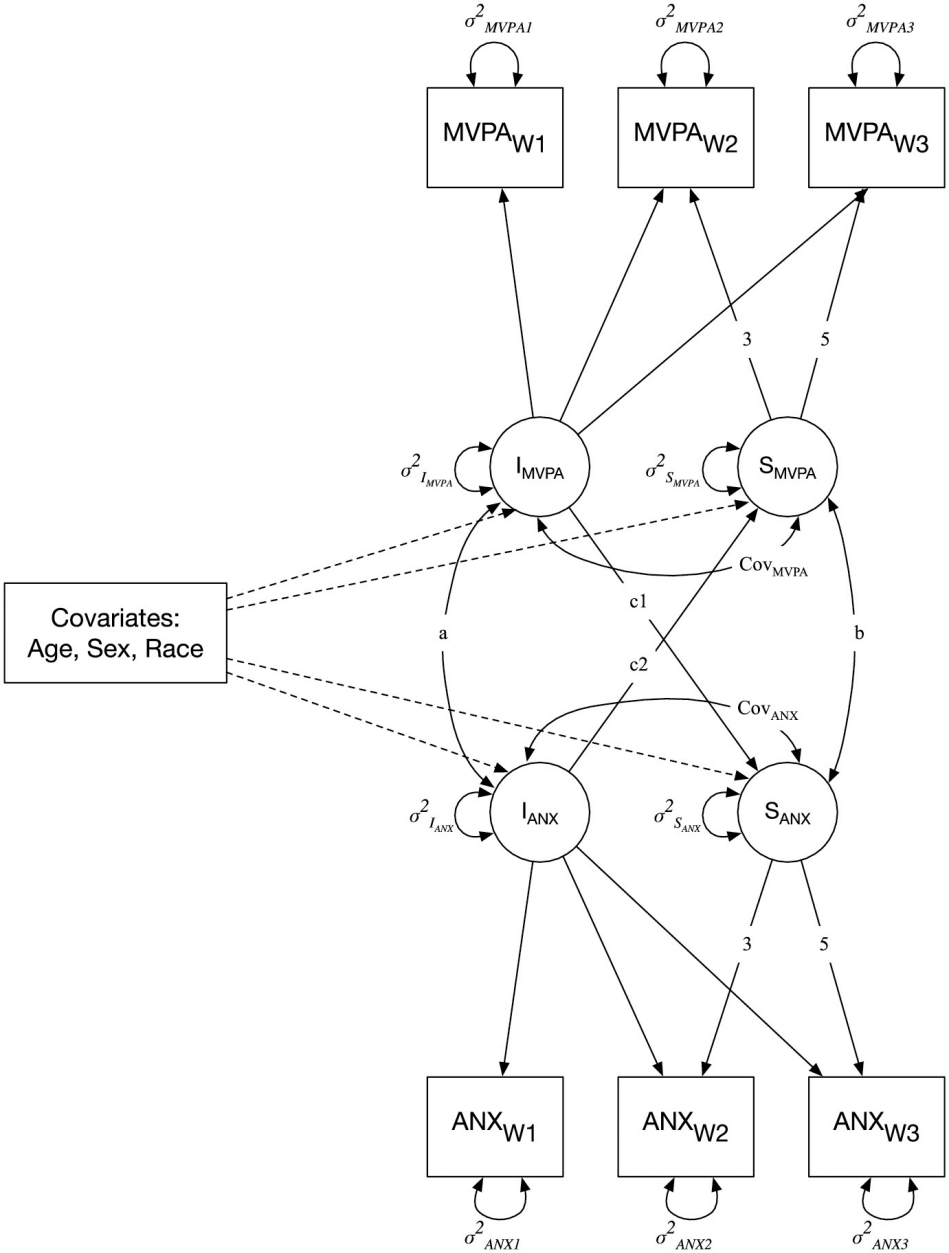
Parallel process latent growth curve model estimating the parallel changes in moderate-to-vigorous physical activity (MVPA) and anxiety from follow-up surveys W1 to W3. I = latent intercept. S = latent slope. Cov = covariance between the latent intercept and slope. MVPA = moderate-to-vigorous-physical activity. ANX = anxiety. W1-W3 = follow-up survey waves 1 to 3. a = cross-sectional association between MVPA and anxiety at W1. b = parallel association between the change in MVPA and change in anxiety from W1 to W3. c1 and c2 = prospective associations between MVPA/anxiety at W1 and change in MVPA/anxiety from W1 to W3. *Note*. The growth parameters are constrained to 0 (illustrated with the lack of arrow), 3, and 5 to illustrate the number of months between W1, W2, and W3.

Next, four parallel process LGCMs were used to examine the associations between activity (MVPA and walking) and mental health outcomes (anxiety and stress). An example illustrating the parallel process between MVPA and anxiety is presented in Fig 2. For each of the two variables (i.e., MVPA and anxiety), a latent intercept (Intercept_PA_ and Intercept_ANX_), a latent slope (Slope_PA_ and Slope_ANX_), and the covariance between the latent intercept and slope (Cov_PA_ and Cov_ANX_) were estimated. As described above, the latent intercept represents the average level of MVPA/anxiety at W1, the latent slope indicates the average change in MVPA/anxiety from W1 to W3, and the covariance between the latent intercept and slope reflects the association between the level of MVPA/anxiety at W1 and corresponding change over time. Three additional parameters specific to the parallel process LGCM were estimated: 1) the cross-sectional association (Cov_(I_PA, I_ANX)_), reflecting the association between MVPA and anxiety levels at W1; 2) the parallel association of change (Cov_(S_PA, S_ANX)_), representing the association of change in MVPA and change in anxiety over time; and 3) the prospective association (Intercept_(PA)_ → Slope_(ANX)_, Intercept_(ANX)_ → Slope_(PA)_), which reflects the extent to which the change in anxiety over time is associated with MVPA at W1 and the change in MVPA is associated with anxiety at W1, respectively.

All LGCMs account for within-twin pair correlations and control for age, sex, and race. However, within-pair analyses were not performed due to limited statistical power given the sample size in the current study. MVPA and walking are modeled as minutes per week in the univariate LGCMs, but modeled as hours per week in the parallel process LGCMs to allow variables to be on similar scales. As the two mental health indicators are highly skewed, anxiety and stress are square root transformed in all models. All statistical models were estimated in Mplus 8.1 [33] using full-information maximum likelihood (FIML) for missing data on MVPA (*n* = 61), walking (*n* = 88), anxiety (*n* = 26), and/or stress (*n* = 27).

## Results

The final analytic sample included *N* = 757, *N* = 563, and *N* = 547 twin pairs for Waves 1, 2, and 3 respectively (Table 1). Average follow-up time was 88.4 ± 4.8 days between Waves 1 and 2, and 63.3 ± 5.8 days between Waves 2 and 3. Across the three survey waves, roughly 30% of the sample were men and 70% were women. The majority of the participants self-identified as White (~96%). The average age of participants was around 52 years, with a range of 20–93 years. Socioeconomic status was not assessed in the present study. However, another recent WSTR COVID-19 related study showed that most participants were in middle to upper class economic categories [5], which is consistent with the larger WSTR demographic profile [23, 34].

**Table 1:** Sample demographic characteristics, physical activity levels, and mental health outcomes over time among twins in the Washington State Twin Registry.

Dates	W1	W2	W3
	Apr 20–May 3	Jul 16–Aug 2	Sept 16–Oct 1
*N*	3057	2608	2491
*N* pairs	757	563	547
Sex			
Male (%)	29.8%	30.3%	30.1%
Female (%)	70.2%	69.7%	69.9%
White (%)	95.7%	96.1%	96.0%
	*M (SD*)	*M (SD*)	*M (SD*)
Age (years)	51.3 (*16*.*0*)	52.5 (*16*.*1*)	52.5 (*16*.*0*)
	Range = 20.1–92.3	Range = 21.0–92.5	Range = 21.3–92.7
*MVPA	124.4 *(97*.*8)*	121.9 *(97*.*5)*	113.8 *(96*.*1)*
	Range = 0.0–350.0	Range = 0.0–350.0	Range = 0.0–350.0
*Walking	173.0 *(142*.*3)*	156.3 *(138*.*7)*	132.9 *(128*.*6)*
	Range = 0.0–630.0	Range = 0.0–630.0	Range = 0.0–630.0
^α^Stress	10.9 *(6*.*9)*	11.0 *(7*.*0)*	11.5 *(7*.*2)*
^β^Anxiety	2.9 *(3*.*2)*	2.8 *(3*.*3)*	3.0 *(3*.*5)*

W1-W3 = follow-up survey waves 1 to 3. *M* = mean. *SD* = standard deviation.

MVPA = moderate-to-vigorous physical activity.

*MVPA and Walking are presented as minutes per week.

^α^Anxiety score ranges from 0 to 24;

^β^Stress score ranges from 0 to 40.

The average MVPA levels remained similar between W1 and W2, with a slight decrease of about 7 minutes per week at W3. The average walking levels showed a slight decline from W1 to W2, with a further decrease of about 25 minutes at W3. Both mental health indicators remained consistently low across W1 to W3.

There was no significant difference in gender between those who completed all three waves versus those who completed one or two waves (*χ*^2^ = .06, *p* = .808). Those who completed all three waves were older (*Est* = 3.41, *SE* = .69, *p* < .001) than those who completed one or two waves. After square-root transformation to correct for skewness of the data, those who completed all three waves were slightly less stressed (*Est* = -.16, *SE* = .05, *p* < .001), with more MVPA (*Est* = .86, *SE* = .32, *p* < .001) and walking (*Est* = 1.10, *SE* = .23, *p* < .001) than those who completed one or two waves. Despite being statistically significant, the values transformed to, on average, less than one point difference in stress, less than 10-minute difference in MVPA, and less than 17-minute difference in walking between those who completed all three waves and those who only completed one or two waves.

As shown in Table 2, correlations between the two physical activity predictors and mental health outcomes were consistently weak across waves (all *r*s < -.15 for MVPA; all *r*s < .16 for walking). Correlations of each measure (MVPA, walking, anxiety, and stress) within individuals over time (S1 Table), and twin pair correlations on the outcomes by zygosity (S2 Table), are included in Supporting Information.

**Table 2:** Correlations between physical activity and mental health outcomes over time.

	W1			W2			W3		
	*r*	95%CI	*p*	*r*	95%CI	*p*	*r*	95%CI	*p*
Associations between MVPA and mental health outcomes									
Anxiety	-.07	[-.11, -.04]	< .001	-.03	[-.07,.01]	.112	-.08	[-.12, -.04]	< .001
Stress	-.11	[-.15, -.08]	< .001	-.12	[-.16, -.08]	< .001	-.14	[-.18, -.10]	< .001
Associations between walking and mental health outcomes									
Anxiety	-.04	[-.07, 0]	.036	-.09	[-.13, -.05]	< .001	-.12	[-.16, -.09]	< .001
Stress	-.11	[-.15, -.08]	< .001	-.16	[-.20, -.12]	< .001	-.16	[-.20, -.12]	< .001

W1-W3 = follow-up survey waves 1 to 3. *r* = Pearson correlations. MVPA = moderate-to-vigorous physical activity.

### Change in physical activity over time

The estimated parameters from the LCGMs investigating the change in physical activity from W1 to W3 are shown in Table 3. The latent intercepts reflect the average initial levels of MVPA (Est = 114.63, *SE* = 2.69) and walking (Est = 150.62, *SE* = 3.78) at W1. The latent slopes represent the average changes in physical activity from W1 to W3; there was a statistically significant decrease in MVPA (Est = -7.26, *SE* = 2.14, *p* = .001) and walking (Est = -20.63, *SE* = 3.02, *p* < .001) over time. On average, there was an approximately seven minutes per week decrease in MVPA and about 20 minutes per week decrease in walking between the W1 and W3 surveys. The covariance between the latent intercept and slope for the MVPA model was not statistically significant (Est = -162.17, *SE* = 86.60, *p* = .061), suggesting that participants’ MVPA levels at W1 were not associated with the amount of change in MVPA over time. On the other hand, the statistically significant covariance between the latent intercept and slope for walking (Est = -1172.79, *SE* = 213.25, *p* < .001) suggests that individuals who reported more walking at W1 had a larger decrease in walking over time, as compared to those who reported less walking at W1.

**Table 3:** Latent growth curve models examining the changes in physical activity and mental health outcomes from follow-up surveys waves 1 to 3.

	Intercept			Slope			Cov_*(I*,*S)*_		
	Est	*SE*	*p*	Est	*SE*	*p*	Est	*SE*	*p*
MVPA	114.63	2.69	< .001	-7.26	2.14	.001	-162.17	86.60	.061
Walking	150.62	3.78	< .001	-20.63	3.02	< .001	-1172.79	213.25	< .001
Anxiety	1.42	.03	< .001	.04	.02	.105	-.03	.01	.003
Stress	3.17	.03	< .001	.06	.02	.008	-.32	.07	< .001

Cov_*(I*,*S)*_ = covariance between the latent intercept (I) and slope (S). Est = unstandardized parameter estimate. *SE* = standard error. MVPA = moderate-to-vigorous activity. *Note*. All models consider within-twin pair correlations and control for age, sex, and race. Anxiety and stress are square root transformed.

### Change in mental health outcomes over time

The estimated latent intercepts reflect the average levels of anxiety and stress at W1. The latent slope for anxiety was not statistically significant (Est = .04, *SE* = .02, *p* = .105), suggesting levels of anxiety remained stable from W1 to W3. The statistically significant latent slope for stress (Est = .06, *SE* = .02, *p* = .008) suggests a small increase in perceived stress over time. There was a statistically significant negative covariance between latent intercept and slope for stress (Est = -.32, *SE* = .07, *p* < .001), suggesting that participants with higher stress levels at W1 had a slower increase in stress over time, compared to those with lower stress levels at W1.

### Parallel change in physical activity and anxiety

Results from the parallel process LCGM estimating the parallel changes in physical activity and anxiety are presented in Table 4. The cross-sectional associations between MVPA and anxiety (Est = -.10, *SE* = .03, *p* = .002) and between walking and anxiety (Est = -.09, *SE* = .04, *p* = .038) were statistically significant, meaning that individuals with more physical activity at W1 were also less anxious at W1. There was no substantial parallel association between changes in either measure of physical activity and anxiety. There was no statistically significant prospective association between MVPA and anxiety, meaning that the average levels of MVPA and anxiety at W1 were not associated with the change in anxiety and MVPA from W1 to W3. There was a small negative prospective association between the amount of walking at W1 and change in anxiety over time (Est = -.05, *SE* = .01, *p* < .001); however, the effect was minimal. The prospective association between the latent intercept of anxiety and latent slope of walking was not statistically significant, suggesting that the average level of anxiety at W1 was not associated with the change in walking over time.

**Table 4:** Unstandardized parameter estimates from the parallel latent growth curve model estimating the changes in physical activity and anxiety over time.

	MVPA			Walking		
	Est	*SE*	*p*	Est	*SE*	*p*
Outcome: PA						
Intercept_(PA)_	2.00	.04	< .001	2.72	.05	< .001
Slope_(PA)_	-.02	.02	.491	-.07	.03	.028
Cov_(PA)_	-.05	.02	.047	-.32	.06	< .001
Outcome: Anxiety						
Intercept_(ANX)_	1.38	.03	< .001	1.37	.03	< .001
Slope_(ANX)_	-.003	.02	.886	-.004	.02	.842
Cov_(ANX)_	-.02	.01	.011	-.02	.01	.013
Cross-sectional association ^a^						
Intercept_(PA)_, Intercept_(ANX)_	-.10	.03	.002	-.09	.04	.038
Parallel association ^b^						
Slope_(PA)_, Slope_(ANX)_	-.001	.001	.141	-.001	.001	.408
Prospective association ^c^						
Intercept_(PA)_ → Slope_(ANX)_	.001	.003	.870	-.05	.01	< .001
Intercept_(ANX)_ → Slope_(PA)_	-.01	.01	.440	-.003	.002	.091

MVPA = moderate-to-vigorous physical activity. Est = unstandardized parameter estimate. *SE* = standard error. PA = physical activity. Cov = covariance. ANX = anxiety.

^a^ Cross-sectional association can be interpreted as changes in baseline MVPA/walking (h/wk) by one square-root unit changes in baseline anxiety.

^b^ Parallel association can be interpreted as changes in the slope of MVPA/walking (h/wk) by one square-root unit changes in the slope of anxiety.

^c^ Prospective association can be interpreted as changes in the slope of MVPA or anxiety by one square-root unit changes in baseline anxiety or MVPA/walking.

*Note*. All models consider within-twin pair correlations and control for age, sex, and race. Anxiety and stress are square root transformed.

### Parallel change in physical activity and stress

The parallel process LCGM estimating the parallel changes in physical activity and stress are shown in Table 5. There was a statistically significant cross-sectional association between MVPA and stress (Est = -.20, *SE* = .03, *p* < .001), suggesting that individuals with more physical activity at W1 had lower perceived stress levels at W1. There was a small negative parallel association between the change in MVPA and change in stress over time (Est = -.003, *SE* = .001, *p* = .001); however, as there was no substantial change in stress levels, this significant result did not have substantive meaning. There was no statistically significant parallel association between walking and stress, meaning that changes in walking from W1 to W3 were not associated with changes in stress over time. There were no significant prospective associations, suggesting that the average levels of physical activity and stress at W1 were not associated with the change in stress and physical activity over time.

**Table 5:** Unstandardized parameter estimates from the parallel latent growth curve model estimating the changes in physical activity and stress over time.

	MVPA			Walking		
	Est	*SE*	*p*	Est	*SE*	*p*
Outcome: PA						
Intercept_(PA)_	2.00	.04	< .001	2.71	.05	< .001
Slope_(PA)_	-.01	.03	.830	-.11	.04	.011
Cov_(PA)_	-.05	.02	.056	-.31	.06	< .001
Outcome: stress						
Intercept_(STRESS)_	3.12	.03	< .001	3.11	.03	< .001
Slope_(STRESS)_	0	.02	.995	-.01	.02	.719
Cov_(STRESS)_	-.03	.01	.032	-.02	.01	.038
Cross-sectional association ^a^						
Intercept_(PA)_, Intercept_(STRESS)_	-.20	.03	< .001	-.32	.05	< .001
Parallel association ^b^						
Slope_(PA)_, Slope_(STRESS)_	-.003	.001	.001	-.002	.001	.106
Prospective association ^c^						
Intercept_(PA)_ → Slope_(STRESS)_	.01	.03	.116	-.01	.01	.278
Intercept_(STRESS)_ → Slope_(PA)_	-.01	.01	.186	.001	.002	.473

MVPA = moderate-to-vigorous physical activity. Est = unstandardized parameter estimate. *SE* = standard error. PA = physical activity. Cov = covariance.

^a^ Cross-sectional association can be interpreted as changes in baseline MVPA/walking (h/wk) by one square-root unit changes in baseline stress.

^b^ Parallel association can be interpreted as changes in the slope of MVPA/walking (h/wk) by one square-root unit changes in the slope of stress.

^c^ Prospective association can be interpreted as changes in the slope of MVPA/walking (h/wk) by one square-root unit changes in baseline stress or MVPA/walking.

*Note*. All models consider within-twin pair correlations and control for age, sex, and race. Anxiety and stress are square root transformed.

## Discussion

The current study expands on previous cross-sectional work by using a sample of twin adults to investigate the association between changes in physical activity and changes in mental health outcomes over a six-month period during the COVID-19 pandemic (April to October, 2020). Over this period of data collection, COVID-19 restrictions and mandates fluctuated across the state of Washington with Phase 2 re-opening beginning in some counties May/June of 2020, and more stringent restrictions (Phase 1) being re-implemented Fall of 2020 due to continued spread of the virus. In line with previous literature during COVID-19 [15, 16], individuals who reported more MVPA and walking reported lower anxiety and stress levels compared to those who reported lower levels of physical activity although the effect was minimal, consistently across the three waves of data. We showed that amount of MVPA and walking decreased over this time period, and individuals reporting more walking at W1 had a larger decrease in walking amount over time. Mental health indicators were mostly low and remained stable over time, although a very small increase in stress was observed. Changes in walking over time were not related to changes in anxiety or stress levels, and changes MVPA was not related to changes in anxiety. The amount of walking at baseline was related to a slight decrease in anxiety over time, after taking into account cross-sectional and parallel correlations between walking and anxiety. These results did not support our hypothesis that changes in physical activity would be associated with changes in mental health outcomes over time. Considering that the cross-sectional associations between physical activity and mental health outcomes were weak in the current sample, and that the mental health outcomes remained relatively stable over time, it is possible that our study did not have enough power to detect any associations between the changes in physical activity and mental health outcomes.

The current findings provided follow-up to our previous study, wherein about 44% of twins reported a perceived decrease in physical activity since the implementation of COVID-19 mitigation strategies in Washington State [20]. Federal guidelines recommend adults participate in 150 minutes of MVPA per week [12], and achieve 10,000 steps (approximately 100 minutes of walking) per day [35], to promote health and well-being. The current study showed that, at W1 (April-May 2020), participating twins were below meeting recommendations for weekly MVPA on average (*M* = 124.4, *SD* = 97.8). While it is not clear whether twins were meeting recommendations for daily walking based on neighborhood minutes of walking per week, reductions in both modes of physical activity over time, even as COVID-19 restrictions were modified throughout 2020, are concerning and consistent with previous literature [17]. It is worth noting that the Pacific Northwest experienced significant hazardous air quality due to wildfire smoke roughly September 7–19, 2020, which may have led to further reductions in physical activity levels in the sample.

Present study findings showed that mental health indicators, including anxiety and stress, were mostly low and remained stable over time among our sample. A slight increase in stress over the six-month study period was observed, though this change is not clinically significant (i.e., less than one unit increase of stress score ranging from 0 to 40). Results indicating low levels of anxiety and stress are contrary to previous studies on mental health concerns during the COVID-19 pandemic. For example, a recent review and meta-analysis of 17 studies reported consistently heightened symptoms of anxiety, stress, and depression resulting from pandemic-related concerns [36]. Furthermore, studies have reported numerous subpopulations that may be most vulnerable to negative changes in mental health outcomes during COVID-19, including those in underdeveloped countries [37, 38], women [5, 37], younger adults (aged 21–40 years) [5, 38], and those with high exposure to news/media [38]. Timing of data collection, age and race/ethnicity of study participants, methods of assessing mental health indicators, and regional variation implementing COVID-19 restrictions may be factors influencing differences in findings across studies. A longitudinal study conducted in China reported that, from the time of initial outbreak to four weeks later, respondents had lower levels of psychological impact, although levels still remained undesirably high, throughout the study period [39]. That said, results of the current study conducted in a sample of primarily White twins of at least modest socioeconomic means, mostly residing in Washington State, may suggest that for some in the U.S., anxiety and stress levels have not necessarily increased over time, but rather leveled off by October 2020, since the initial COVID-19 shutdown in March. Given these findings and limitations, future studies should seek to analyze changes in physical activity and stress among those communities most vulnerable to the negative health impacts of COVID-19, including minorities and low-income households [40], where stress and anxiety is likely higher, and activity levels lower, than in the current sample.

The present study did not support the hypothesis that changes in physical activity would be associated with changes in mental health outcomes over time. At each of three waves of data collection over the course of 2020 and during fluctuating COVID-19 mandates, we observed weak negative relationships between physical activity and mental health outcomes. These findings provide a longitudinal follow-up to our baseline study conducted in March 2020, which reported that a perceived decrease in physical activity was related to higher levels of stress and anxiety, whereas a perceived increase in physical activity was not associated with mental health outcomes [20]. The current study adds to the existing literature that, even among a sample of individuals of low distress during the COVID-19 pandemic, those who reported less physical activity were slightly more stressed and more anxious than those who reported more physical activity. Maintaining healthful levels of physical activity while social distancing has been promoted by the Centers for Disease Control and Prevention (CDC) as a necessary means to lower blood pressure, improve mood and stress levels, and achieve better quality of sleep in the context of the pandemic [41]. For these reasons, the CDC recommends at-home strategies to improve physical activity, including active family play time, catching up on household chores that require walking, spending time outside and exercising while watching television. The current study findings combined with previous literature during COVID-19 [15, 16] support that such activities, particularly those performed at moderate-intensity and higher, may have efficacy to combat pandemic-related stressors.

### Strengths and limitations

A primary strength of the present study is its timeliness, specifically that data collection began immediately after the Washington state “stay home, stay healthy” mandate. Thus, the immediate and longitudinal impact of physical distancing measures were captured in accordance with the timeline of these mitigation strategies in a large statewide cohort. Terminology of survey items assessing physical activity behavior and mental health outcomes were tailored to accommodate the quickly changing nature of COVID-19. Specifically, use of “In a typical week” was changed to include, “Thinking about the last two weeks, in a typical week” while data were collected at multiple time points. Finally, the longitudinal nature of this study allowed us to assess how physical activity and mental health outcomes have changed over a six-month period during the COVID-19 pandemic, as well as the extent to which indicators of physical activity and mental health changes affect one another over time. The use of LGCM allowed us to account for inter-person variability when examining changes in each outcome variable over time; the parallel LCGM integrated two LGCMs for each outcome variable in order to produce unbiased estimated of the cross-sectional, prospective, and parallel associations between physical activity and mental health outcomes.

This study is not without limitations. First, the impact of physical distancing measures may be difficult to assess, given wide variation in how such measures were implemented by county in Washington and across states, and individual’s adherence to such recommendations and requirements. As with any survey study design, self-reported behavioral measures are subject to recall bias and social desirability, and most survey instruments are only weakly or moderately correlated with objective measures. Despite the inherent limitations in using self-report activity instruments, our measures were at least moderately correlated with objective activity measures, thus providing support for the use of our instrument in the present study. As discussed in the previous cross-sectional study [20], the sample may have been biased in that those experiencing lower anxiety/stress may have been more likely to participate in the survey. Additionally, there was low retention from baseline to W3 overall (65.7% of individuals, 60.1% of pairs), and those with lower anxiety/stress may have been less likely to drop out of the study over time. For these reasons, and due to the sample being primarily White and female (95.5% and 70.2% at baseline, respectively), generalizability of the study findings is limited. Seasonality of data collection may have independently influenced levels of physical activity and mental health outcomes. The WSTR relies on the voluntary participation of twins, which could lead to selection bias, as evidenced by our large proportion of identical and female twins. Relatedly, participation in this study was limited to twins who had previously completed the baseline survey, which could lead to additional selection bias. It is unclear whether twins who have more positive mental health outcomes are more likely to participate in the current study; it is also unclear whether the associations between physical activity and mental health outcomes differ among the non-participants. Finally, within-pair twin analyses were not performed due to limited sample size. It remains unclear whether the longitudinal associations between physical activity and mental health outcomes are mediated by between-family confounds.

## Conclusions

In conclusion, physical activity was related to mental health outcomes cross-sectionally at each of three waves of data collection. Findings suggest that changes in MVPA, but not walking, are negatively associated with changes in stress levels during COVID-19, though the effect was minimal in this sample with relatively low stress. From April to October 2020, levels of physical activity decreased whereas mental health outcomes generally remained stable in this sample. Overall, the current study results and previous literature support efforts of public health practitioners and the CDC to promote physical activity as a means to improve health and well-being of individuals during implementation of social distancing restrictions. Moving forward, as vaccines to combat COVID-19 become publicly available and social distancing restrictions continue to lift, it remains necessary to assess inevitable long-term outcomes as a result of pandemic-related trauma, and determinants of our communities’ physical and mental health.

## Supporting information

S1 Table Correlations within individuals over time. (DOCX)

S2 Table Correlations for physical activity and mental health outcomes. (DOCX)

## References

[1] Hui DS, Azhar EI, Madani TA, Ntoumi F, Kock R, Dar O, et al. The continuing 2019-nCoV epidemic threat of novel coronaviruses to global health—The latest 2019 novel coronavirus outbreak in Wuhan, China. Int J Infect Dis. 2020;91:264–6. doi: 10.1016/j.ijid.2020.01.009 31953166 PMC7128332

[2] World Health Organization. WHO Coronavirus Disease (COVID-19) Dashboard. Geneva: World Health Organization; 2020.

[3] Anderson RM, Heesterbeek H, Klinkenberg D, Hollingsworth TD. How will country-based mitigation measures influence the course of the COVID-19 epidemic? Lancet. 2020;395(10228):931–4. doi: 10.1016/S0140-6736(20)30567-5 32164834 PMC7158572

[4] Sen-Crowe B, McKenney M, Elkbuli A. Social distancing during the COVID-19 pandemic: Staying home save lives. Am J Emerg Med. 2020;38(7):1519–20. doi: 10.1016/j.ajem.2020.03.063 32305155 PMC7194642

[5] Tsang S, Avery AR, Duncan GE. Fear and depression linked to COVID-19 exposure A study of adult twins during the COVID-19 pandemic. Psych Res. 2021;296:113699. doi: 10.1016/j.psychres.2020.113699 33401090 PMC7772100

[6] Hallam K, Bilsborough S, de Courten M. “Happy feet”: evaluating the benefits of a 100-day 10,000 step challenge on mental health and wellbeing. BMC Psych. 2018;18(1):1–7. doi: 10.1186/s12888-018-1609-y 29361921 PMC5781328

[7] Bernard P, Doré I, Romain A-J, Hains-Monfette G, Kingsbury C, Sabiston C. Dose response association of objective physical activity with mental health in a representative national sample of adults: A cross-sectional study. PLoS One. 2018;13(10):e0204682. doi: 10.1371/journal.pone.0204682 30356252 PMC6200189

[8] Schultchen D, Reichenberger J, Mittl T, Weh TR, Smyth JM, Blechert J, et al. Bidirectional relationship of stress and affect with physical activity and healthy eating. Br J Health Psychol. 2019;24(2):315–33. doi: 10.1111/bjhp.12355 30672069 PMC6767465

[9] McDowell CP, Dishman RK, Gordon BR, Herring MP. Physical activity and anxiety: a systematic review and meta-analysis of prospective cohort studies. Am J Prev Med. 2019;57(4):545–56. doi: 10.1016/j.amepre.2019.05.012 31542132

[10] Ströhle A. Physical activity, exercise, depression and anxiety disorders. J Neural Transm. 2009;116(6):777–84. doi: 10.1007/s00702-008-0092-x 18726137

[11] Tables of summary health statistics for US adults: 2018 National Health Interview Survey. [Internet]. National Center for Health Statistics. 2019 [cited February 11, 2021]. https://ftp.cdc.gov/pub/Health_Statistics/NCHS/NHIS/SHS/2018_SHS_Table_A-14.pdf.

[12] Piercy KL, Troiano RP, Ballard RM, Carlson SA, Fulton JE, Galuska DA, et al. The Physical Activity Guidelines for Americans. JAMA. 2018;320(19):2020–8. doi: 10.1001/jama.2018.14854 30418471 PMC9582631

[13] Althoff T, Hicks JL, King AC, Delp SL, Leskovec J. Large-scale physical activity data reveal worldwide activity inequality. Nature. 2017;547(7663):336–9. doi: 10.1038/nature23018 28693034 PMC5774986

[14] Vogel EA, Zhang JS, Peng K, Heaney CA, Lu Y, Lounsbury D, et al. Physical activity and stress management during COVID-19: a longitudinal survey study. Psychol Health. 2020:1–11. 10.1080/08870446.2020.1869740 33405969

[15] Woodruff SJ, Coyne P, St-Pierre E. Stress, physical activity, and screen-related sedentary behaviour within the first month of the COVID-19 pandemic. Appl Psychology: Health Well-Being. 2021;13(2):454–68. doi: 10.1111/aphw.12261 33645893 PMC8014671

[16] Stanton R, To QG, Khalesi S, Williams SL, Alley SJ, Thwaite TL, et al. Depression, anxiety and stress during COVID-19: associations with changes in physical activity, sleep, tobacco and alcohol use in Australian adults. Int J Env Res Public Health. 2020;17(11):4065. 10.3390/ijerph17114065 PMC7312903 32517294

[17] Pellegrini M, Ponzo V, Rosato R, Scumaci E, Goitre I, Benso A, et al. Changes in weight and nutritional habits in adults with obesity during the “lockdown” period caused by the COVID-19 virus emergency. Nutrients. 2020;12(7):2016. 10.3390/nu12072016 PMC7400808 32645970

[18] Zachary Z, Brianna F, Brianna L, Garrett P, Jade W, Alyssa D, et al. Self-quarantine and weight gain related risk factors during the COVID-19 pandemic. Obes Res Clin Pract. 2020;14(3):210–6. doi: 10.1016/j.orcp.2020.05.004 32460966 PMC7241331

[19] Bhutani S, Cooper JA. COVID-19–Related Home Confinement in Adults: Weight Gain Risks and Opportunities. Obesity. 2020;28(9):1576–7. doi: 10.1002/oby.22904 32428295 PMC7276847

[20] Duncan GE, Avery AR, Seto E, Tsang S. Perceived change in physical activity levels and mental health during COVID-19: Findings among adult twin pairs. PLoS One. 2020;15(8):e0237695. doi: 10.1371/journal.pone.0237695 32790745 PMC7425865

[21] Turkheimer E, Harden KP. Behavior genetic research methods. In: Reis HT, Judd CM, editors. Handbook of Research Methods in Social and Personality Psychology. Cambridge: Cambridge University Press; 2014. pp. 159–87.

[22] Afari N, Noonan C, Goldberg J, Edwards K, Gadepalli K, Osterman B, et al. University of Washington Twin Registry: construction and characteristics of a community-based twin registry. Twin Res Hum Genet. 2006;9(6):1023–9. doi: 10.1375/183242706779462543 17254446 PMC2953369

[23] Duncan GE, Avery AR, Strachan E, Turkheimer E, Tsang S. The Washington State Twin Registry: 2019 Update. Twin Res Hum Genet. 2019;22(6):788–93. doi: 10.1017/thg.2019.36 31358074

[24] Strachan E, Hunt C, Afari N, Duncan G, Noonan C, Schur E, et al. University of Washington Twin Registry: Poised for the Next Generation of Twin Research. Twin Res Human Genet. 2012;16(1):455–62. doi: 10.1017/thg.2012.124 23218177 PMC4505360

[25] Patridge EF, Bardyn TP. Research electronic data capture (REDCap). J Med Lib Assoc: JMLA. 2018;106(1):142.

[26] Garber CE, Blissmer B, Deschenes MR, Franklin BA, Lamonte MJ, Lee IM, et al. American College of Sports Medicine position stand. Quantity and quality of exercise for developing and maintaining cardiorespiratory, musculoskeletal, and neuromotor fitness in apparently healthy adults: guidance for prescribing exercise. Med Sci Sports Exerc. 2011;43(7):1334–59. doi: 10.1249/MSS.0b013e318213fefb 21694556

[27] U.S. Department of Health and Human Services. Physical activity and health: a report of the Surgeon General. Atlanta: U.S. Department of Health and Human Services, Centers for Disease Control and Prevention, National Center for Chronic Disease Prevention and Health Promotion, 1996.

[28] Lee C, Moudon AV. Environmental correlates of walking for transportation or recreation purposes. J Phys Act Health. 2006;Suppl 1:S77–98. 10.1123/jpah.3.s1.s77 28834524

[29] Moudon AV, Lee C, Cheadle AD, Garvin C, Johnson D, Schmid TL, et al. Operational Definitions of Walkable Neighborhood: Theoretical and Empirical Insights. J Phys Act Health. 2006;3(s1):S99–S117. doi: 10.1123/jpah.3.s1.s99 28834523

[30] Derogatis LR, Melisaratos N. The Brief Symptom Inventory: an introductory report. Psychol Med. 1983;13(3):595–605. 6622612

[31] Cohen S, Kamarck T, Mermelstein R. A global measure of perceived stress. J Health Soc Behav. 1983;24(4):385–96. 6668417

[32] Preacher KJ, Wichman AL, MacCallum RC, Briggs NE. Latent growth curve modeling. In: Liao TF, editor. Thousand Oaks, CA: Sage Publications; 2008.

[33] Muthén LK, Muthén B. Mplus. Statistical analysis with latent variables. User’s Guide. 7th ed. Los Angeles, CA: Muthen & Muthen; 2012.

[34] Duncan GE, Avery A, Hurvitz PM, Moudon AV, Tsang S, Turkheimer E. Cohort Profile: TWINS study of environment, lifestyle behaviours and health. Int J Epidemiol. 2018;48(4):1041–h. 10.1093/ije/dyy224 PMC6693812 30428089

[35] Tudor-Locke C, Bassett DR. How many steps/day are enough? Preliminary pedometer indices for public health. Sports Med. 2004;34. doi: 10.2165/00007256-200434010-00001 14715035

[36] Salari N, Hosseinian-Far A, Jalali R, Vaisi-Raygani A, Rasoulpoor S, Mohammadi M, et al. Prevalence of stress, anxiety, depression among the general population during the COVID-19 pandemic: a systematic review and meta-analysis. Globalization Health. 2020;16(1):1–11. 32631403 10.1186/s12992-020-00589-w PMC7338126

[37] Zhou S-J, Zhang L-G, Wang L-L, Guo Z-C, Wang J-Q, Chen J-C, et al. Prevalence and socio-demographic correlates of psychological health problems in Chinese adolescents during the outbreak of COVID-19. Eur Child Adoles Psy. 2020;29(6):749–58. doi: 10.1007/s00787-020-01541-4 32363492 PMC7196181

[38] Moghanibashi-Mansourieh A. Assessing the anxiety level of Iranian general population during COVID-19 outbreak. Asian J Psy. 2020;51:102076. doi: 10.1016/j.ajp.2020.102076 32334409 PMC7165107

[39] Wang C, Pan R, Wan X, Tan Y, Xu L, McIntyre RS, et al. A longitudinal study on the mental health of general population during the COVID-19 epidemic in China. Brain Behav Immun. 2020;87:40–8. doi: 10.1016/j.bbi.2020.04.028 32298802 PMC7153528

[40] Purtle J. COVID-19 and mental health equity in the United States. Social Psych Psych Epid. 2020;55(8):969–71. doi: 10.1007/s00127-020-01896-8 32556376 PMC7298157

[41] How to Be Physically Active While Social Distancing. Centers for Disease Control and Prevention: Division of Nutrition, Physical Activity, and Obesity; 2021 [cited 2021 February 26]. https://www.cdc.gov/physicalactivity/how-to-be-physically-active-while-social-distancing.html.

