# Supplementary material for: Changes in physical activity levels and mental health during COVID-19: Prospective findings among adult twin pairs
Source: PLoS One. 2021 Nov 22;16(11):e0260218. doi: 10.1371/journal.pone.0260218 (PMC8608318; doi:10.1371/journal.pone.0260218)
Supplement: S1 Table — (DOCX) [file pone.0260218.s001.docx]

Table A1. Correlations within individuals over time

|  | W1 & W2 | | | W1 & W3 | | | W2 & W3 | | |
| --- | --- | --- | --- | --- | --- | --- | --- | --- | --- |
|  | *r* | 95%CI | *p* | *r* | 95%CI | *p* | *r* | 95%CI | *p* |
| MVPA | .61 | [.58 - .63] | <.001 | .58 | [.56-.61] | <.001 | .65 | [.62-.67] | <.001 |
| Walking | .66 | [.64 - .68] | <.001 | .58 | [.55-.61] | <.001 | .71 | [.69-.73] | <.001 |
| Anxiety | .73 | [.71-.75] | <.001 | .69 | [.66-.71] | <.001 | .76 | [.74-.77] | <.001 |
| Stress | .74 | [.72-.76] | <.001 | .72 | [.70-.74] | <.001 | .77 | [.75-.79] | <.001 |

W1-W3 = follow-up survey waves 1 to 3. *r* = Pearson correlations. MVPA = moderate-to-vigorous physical activity.
