# Supplementary material for: Changes in physical activity levels and mental health during COVID-19: Prospective findings among adult twin pairs
Source: PLoS One. 2021 Nov 22;16(11):e0260218. doi: 10.1371/journal.pone.0260218 (PMC8608318; doi:10.1371/journal.pone.0260218)
Supplement: S2 Table — (DOCX) [file pone.0260218.s002.docx]

Table A2. Twin correlations for physical activity and mental health outcomes.

|  |  | MZ | | | DZ | | |
| --- | --- | --- | --- | --- | --- | --- | --- |
|  |  | *r* | 95%CI | *p* | *r* | 95%CI | *p* |
| MVPA | W1 | .27 | [.19, .34] | <.001 | .14 | [.01, .26] | .03 |
|  | W2 | .35 | [.25, .44] | <.001 | .19 | [.05, .33] | .01 |
|  | W3 | .31 | [.21, .40] | <.001 | .12 | [-.03, .27] | .11 |
| Walking | W1 | .31 | [.23, .39] | <.001 | .21 | [.08, .33] | <.001 |
|  | W2 | .32 | [.23, .40] | <.001 | .12 | [-.02, .26] | .10 |
|  | W3 | .35 | [.26, .44] | <.001 | .14 | [-.01, .28] | .07 |
| Anxiety | W1 | .40 | [.33, .47] | <.001 | .19 | [.07, .31] | <.001 |
|  | W2 | .41 | [.32, .49] | <.001 | .24 | [.10, .37] | <.001 |
|  | W3 | .29 | [.19, .38] | <.001 | .24 | [.10, .38] | <.001 |
| Stress | W1 | .49 | [.42, .55] | <.001 | .19 | [.07, .31] | <.001 |
|  | W2 | .44 | [.35, .51] | <.001 | .27 | [.13, .40] | <.001 |
|  | W3 | .42 | [.33, .50] | <.001 | .25 | [.10, .38] | <.001 |

MZ = monozygotic twins. DZ = dizygotic twins. W1-W3 = follow-up survey waves 1 to 3. *r* = Pearson correlations. MVPA = moderate-to-vigorous physical activity.
